# *Centella asiatica* Promotes Antioxidant Gene Expression and Mitochondrial Oxidative Respiration in Experimental Autoimmune Encephalomyelitis

**DOI:** 10.3390/ph17121681

**Published:** 2024-12-13

**Authors:** Payel Kundu, Kanon Yasuhara, Mikah S. Brandes, Jonathan A. Zweig, Cody J. Neff, Sarah Holden, Kat Kessler, Steven Matsumoto, Halina Offner, Carin S. Waslo, Arthur Vandenbark, Amala Soumyanath, Larry S. Sherman, Jacob Raber, Nora E. Gray, Rebecca I. Spain

**Affiliations:** 1 Department of Behavioral Neuroscience, Oregon Health & Science University (OHSU), Portland, OR 97239, USA; 2 Division of Neuroscience, Oregon National Primate Research Center, Oregon Health & Science University (OHSU), Beaverton, OR 97006, USA; 3 Department of Neurology, Oregon Health & Science University (OHSU), Portland, OR 97239, USA; 4 BENFRA Botanical Dietary Supplements Research Center, Oregon Health & Science University (OHSU), Portland, OR 97239, USA; 5 Department of Integrative Biosciences, Oregon Health & Science University (OHSU), Portland, OR 97239, USA; 6 Neurology Division, Portland VA Medical Center, Portland, OR 97239, USA; 7 Department of Anesthesiology and Perioperative Medicine, Oregon Health & Science University (OHSU), Portland, OR 97239, USA; 8 Department of Molecular Microbiology and Immunology, Oregon Health & Science University (OHSU), Portland, OR 97239, USA; 9 Department of Cell, Developmental and Cancer Biology, Oregon Health & Science University (OHSU), Portland, OR 97239, USA; 10 Department of Radiation Medicine, Oregon Health & Science University (OHSU), Portland, OR 97239, USA

**Keywords:** *Centella asiatica*, antioxidant, experimental autoimmune encephalomyelitis, mitochondrial respiration

## Abstract

**Background/Objectives:** *Centella asiatica* (L.) Urban (family Apiaceae) (*C. asiatica*) is a traditional botanical medicine used in aging and dementia. Water extracts of *C. asiatica* (CAW) have been used to treat neuropsychiatric symptoms in related animal models and are associated with increases in antioxidant response element (ARE) genes and improvements in mitochondrial respiratory function and neuronal health. Because multiple sclerosis (MS) shares its neurogenerative pathology of oxidative stress and mitochondrial dysfunction with aging and dementia, neuropsychiatric symptoms in MS may also benefit from *C. asiatica.* To determine whether CAW similarly benefits neuropsychiatric symptoms, ARE gene expression, and mitochondrial respiration in inflammatory models of MS, and to determine the effects of CAW on clinical disability and inflammation, we tested CAW using experimental autoimmune encephalomyelitis (EAE). **Methods:** C57BL/6J mice induced with EAE were treated with CAW or a placebo for 2 weeks. The outcomes were clinical disability, signs of anxiety (open field test), ARE gene expression, mitochondrial respiration, and inflammation and demyelination. **Results:** At the dosing schedule and concentrations tested, CAW-treated mice with EAE demonstrated increased ARE gene expression and mitochondrial respiratory activity compared to those of placebo-treated mice with EAE. CAW was also associated with reduced inflammatory infiltrates in the spinal cord, but the differences between the populations of activated versus quiescent microglia were equivocal. CAW did not improve behavioral performance, EAE motor disability, or demyelination. **Conclusions:** In the inflammatory EAE model of MS, CAW demonstrates similar neuroprotective effects to those it exhibits in aging and dementia mouse models. These benefits, along with the anti-inflammatory effects of CAW, support further investigation of its neuropsychiatric effects in people with MS.

## 1. Introduction

Neurological and neuropsychiatric symptoms in multiple sclerosis (MS), an autoimmune neuroinflammatory disorder of the central nervous system (CNS), are frequent and challenging to treat. The most common neurological symptom is cognitive dysfunction, with prevalence ranging from 34–91%, and the most common neuropsychiatric symptoms are depression and anxiety, occurring at rates three to ten times those of the general population [1]. These disabling symptoms often co-occur, start early, worsen in frequency and severity over time, and are more prominent in progressive MS subtypes [2,3]. The impaired cognitive domains include information processing speed, episodic verbal and visuo-spatial memory, verbal fluency, working memory, and executive dysfunction.

Treatment of MS psychiatric symptoms consists of standard behavioral therapies and medications, while treatment of MS-related cognitive dysfunction remains particularly elusive. Current MS disease-modifying therapies (DMT), while highly effective at reducing neuroinflammatory attacks, have only a small to moderate positive effect on MS cognition, as shown in a meta-analysis of 41 studies involving 7131 patients [4]. This study identified no differences in cognitive benefit between lower-efficacy and higher-efficacy DMT, suggesting that non-inflammatory neurodegenerative pathological processes form the basis of MS cognitive dysfunction. The Food and Drug Administration (FDA)-approved dementia medications donepezil and memantine failed to reduce cognitive dysfunction in MS trials, as did the popular supplement *Gingko biloba* L. (family Ginkgoaceae) [5,6,7]. Cognitive behavioral therapy suffers from limited access due to lack of proximity to services, lack of health insurance coverage, and lack of time to participate [8]. Because MS shares pathological processes such as mitochondrial dysfunction and oxidative stress (OS) with other neurodegenerative diseases, effective therapies used in other neurodegenerative diseases that target those processes may also benefit MS cognitive dysfunction [9].

*Centella asiatica* (L.) Urban (family Apiaceae) (*C. asiatica*), a traditional botanical medicine used to support age-related cognitive health, is under active investigation as a treatment for dementia. Preclinical studies using various formulations of *C. asiatica* have shown cognitive benefits in dementia animal models, including the use of a lyophilized powder in rats with streptozotocin-induced dementia and the use of isolated triterpenoid constituent asiaticoside in Aβ_1–42_-induced mice [10,11]. Our group and others have shown that a standardized water extract of *C. asiatica* (CAW) reveals remarkable cognitive behavior-enhancing and neuroprotective properties in animal models of Alzheimer’s and age-induced dementia [12,13]. The mechanisms of CAW associated with clinical benefits are increased antioxidant response element (ARE) gene expression via nuclear factor (erythroid-derived 2)-like 2 (NRF2) regulated pathways, improved mitochondrial respiration, and heightened dendritic arborization, and activation of nuclear factor NRF2 regulated pathways [14,15,16].

The existing clinical trials, despite being few to date, suggest that *C. asiatica* can improve memory and reduce anxiety. Tiwari et. al. conducted an open-label trial where 500 mg capsules of *C. asiatica* extract or placebo were administered twice daily for six months to 60 participants over 65 years with mild cognitive impairment. Pre- to post-treatment mini mental state examination scores significantly improved, as did the proportion of participants reporting feelings of well-being and sensory discomfort [17]. A randomized controlled trial using doses of *C. asiatica* extract ranging from 250 mg to 750 mg, applied daily for two months to 28 elderly participants, resulted in improved working memory, alertness, and calmness [18]. An open-label trial of a hydro-ethanolic extract of *C. asiatica* improved symptoms of generalized anxiety in 33 participants [19]. While these studies are limited variably by small sample sizes, inadequate descriptions of the investigational products, and lack of placebo controls, they encourage further exploration of the clinical benefit of *C. asiatica.*

Because, to date, preclinical and clinical studies of *C. asiatica* have used aging and dementia populations, the potential for benefit in MS is unclear. In this study, we tested the effects of CAW on clinical and mechanistic outcomes in an animal model of MS—specially, experimental autoimmune encephalomyelitis (EAE). Mice with EAE mimic some clinical aspects of MS in humans, including cognitive, behavioral, motor, and sensory deficits, and share pathological aspects of neurodegeneration with aging and dementia models, like oxidative stress and mitochondrial dysfunction [20]. However, unlike those models, EAE has pathophysiological signatures of neuroinflammation including inflammatory infiltrates, microglial activation, and demyelination, which might overshadow neuroprotective benefits [21,22]. The objectives of this study were to test the effects of *C. asiatica* on clinical disability, neuroinflammation, neuropsychiatric symptoms, ARE gene expression, and mitochondrial respiratory activity in mice with EAE. Because EAE causes hind leg paralysis that could interfere with performance in cognitive tests, we focused on measuring anxiety in this initial investigation. 

## 2. Results

### 2.1. Safety and Tolerability

We tested the effects of *C. asiatica* in EAE on clinical, antioxidant, and neuroinflammatory outcomes. In two independent experiments, following induction with EAE, C57BL/6J mice were exposed to either a standardized water extract of *C. asiatica* (CAW) or a placebo in a treatment paradigm that started on day six and lasted for two weeks. Study 1 consisted of two groups, both induced with EAE, while Study 2 added a third group receiving sham EAE and placebo treatment to represent a healthy control population. 

In Study 1, of the 20 mice, 18 received treatment with CAW or a placebo (*n* = 9 per group), as two animals did not reach a clinical disability score of two in sufficient time to receive treatment. Because of the pre-planned sacrifice dates, there were intervals ranging from one to five days between the 14th day of treatment and the day of sacrifice. One placebo-treated mouse received 12 days of treatment instead of 14. In Study 2, all 30 mice received 14 days of treatment up to and including the day of sacrifice. There was no difference in mean weight change between the treatment groups in Study 1 (0.66 g SEM 0.44 vs. 0.28 g SEM 0.38, *p* = 0.26). However, there was an effect of group on body weight in Study 2, such that both EAE cohorts declined in body weight over the study, while the non-EAE control group’s weight stayed constant (*F*(2,27) = 3.350, *p *= 0.050; Tukey’s post hoc control vs. CAW: *p *= 0.040), and there was no difference between the changes in mean weight of the CAW and placebo cohorts.

### 2.2. Clinical Disability, Inflammation, and Demyelination

**Figure 1:**
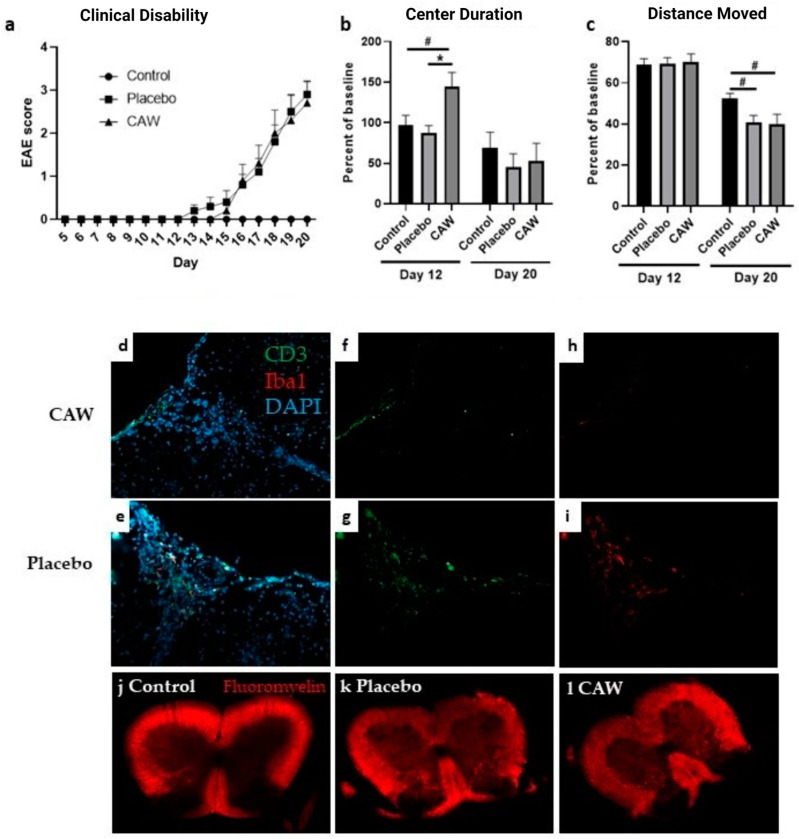
(**a**) Onset and final clinical disability in CAW-treated mice and placebo-treated mice following induction of experimental autoimmune encephalopathy (EAE) were similar between groups in Study 2. Control mice without EAE did not exhibit clinical disability. (**b**) Center Duration as percent of baseline was greater in CAW-treated mice (less anxiety) than placebo or control cohorts at day 12 (early symptomatic), but not at Study 2’s end. (**c**) Distance Moved (activity) in Study 2 day 20 was lower in both CAW and placebo groups compared to controls, likely reflecting disability caused by EAE. (**d**,**e**) Composite stains for CD3 (green, lymphocytes) and Iba1 (red, activated microglia) are shown with DAPI (blue, DNA) in CAW-treated and placebo-treated EAE mouse sections. (**f**,**g**) Decreased CD3 staining in CAW-treated EAE mouse compared to placebo-treated EAE mouse (2.9% STD 1.6% vs. 5.0% STD 1.7%, *p* = 0.01). (**h**,**i**) Trend toward decreased Iba1 staining for activated microglia between CAW- and placebo-treated mice (3.3% STD 2.8% vs. 5.2% STD 5.1%, *p* = 0.30). (**j**–**l**) While both EAE spinal cord sections demonstrate demyelination compared to control, there was no qualitative difference between placebo and CAW. * *p* < 0.05, ^#^ *p* < 0.10.

The mean cumulative disease index (CDI, see Section 4.5), a global measure of motor function, did not differ between the CAW and placebo groups in either study. While the onset of mean clinical disability in the CAW-treated mice in Study 2 (day 15) occurred two days after the mean onset in the placebo-treated mice (day 13), disability equalized by day 16 and remained similar in both cohorts until the study’s end (Figure 1a). 

**Figure 2:**
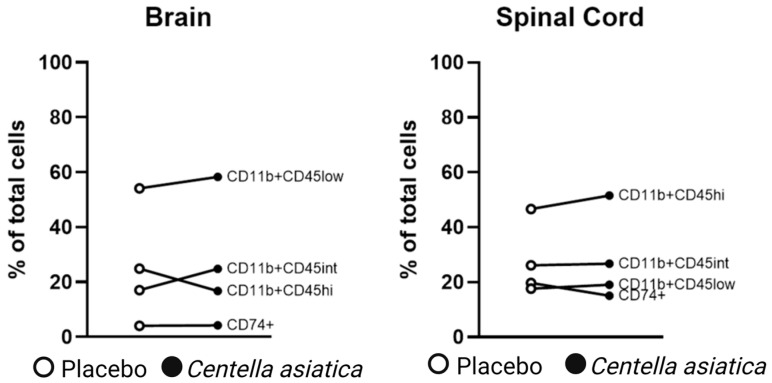
Fluorescence-activated cell sorting analysis of pooled spinal cord samples of monocyte expression (CD74+) resting (CD11b + CD45low) and activated (CD11b + CD45hi) microglia in brains and spinal cords of mice with experimental autoimmune encephalomyelitis (EAE) treated with *Centella asiatica* or placebo. Results presented as percentages (%) of total cell count.

Qualitatively, fluorescence-activated cell sorting (FACS) analysis of pooled spinal cord samples of the three CAW-treated mice in Study 1 demonstrated decreased monocyte (CD74+) expression compared to the three placebo-treated mice (15.0% v 19.7%), but similar expression was found in the pooled brain samples (Table 1, Figure 2). Conversely, a more favorable activated (CD11bCD45hi) to resting (CD11bCD45lo) microglial profile was noted in CAW-treated brains (16.7%/58.3%) vs. placebo (24.9%/54.1%), but not in the CAW-treated spinal cords (51.5%/19.0%) vs. placebo (46.6%/17.7%).

Immunohistochemistry (IHC) of the lumbar spinal cords of the nine CAW-treated mice in Study 2 revealed decreased CD3 counts normalized to total cell counts compared to the ten placebo-treated mice (2.9% STD 1.6% vs. 5.0% STD 1.7%, *p* = 0.01, Figure 1: f&g). Normalized Iba1 counts in the lumbar spinal cords among the ten CAW-treated mice demonstrated a trend towards reduced microglial activation, but this was not different from the nine placebo mice (3.3% STD 2.8% vs. 5.2% STD 5.1%, *p* = 0.30, Figure 1: h&i). There were no qualitative differences in the amounts of demyelination between the mice treated with CAW vs. placebo (Figure 1: k&l). 

### 2.3. Behavioral Performance

Overall, CAW did not impact behavioral performance in Study 1, including Distance Moved, Center Duration, and Latency to First Center Entry in the open field test described in Section 4.5. In Study 2, the Center Duration times on day 12 (early symptomatic) were longer in the CAW-treated mice (*F*(2,27) = 5.229, *p *= 0.012; Tukey’s post hoc: *p* = 0.014 CAW vs. placebo, *p* = 0.050 CAW vs. control), a finding that was not found again for the remainder of the experiment (Figure 1b). An effect of the group on Distance Moved was noted at the study’s end (*F*(2,27) = 3.433, *p *= 0.047); however, this was driven by EAE and not by CAW treatment (Figure 1c). Tukey’s post hoc pairwise comparisons did not reach significance for control vs. CAW (*p* = 0.064) or control vs. placebo (*p* = 0.097). The group had no effect on the Latency to First Center Entry or on Center Frequency.

### 2.4. ARE Gene Expression and Mitochondrial Respiratory Activity

**Figure 3:**
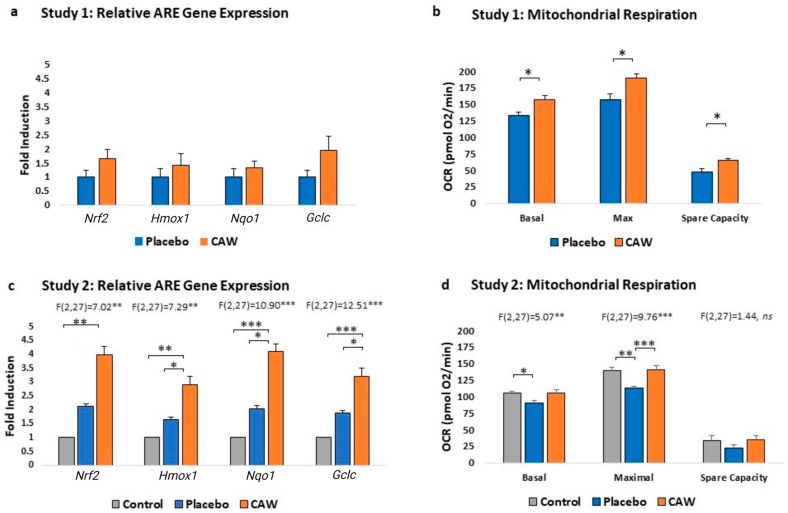
Group comparisons of antioxidant response element (ARE) gene expression and mitochondrial respiration, measured as oxygen consumption rate (OCR), results in Studies 1 (*n* = 4 per group, (**a**,**b**)) and 2 (*n* = 10 per group, (**c**,**d**)). While ARE gene expression in cerebral cortices of CAW-treated mice with experimental autoimmune encephalomyelitis (EAE) was not greater than placebo (**a**), OCR was greater in CAW-treated mice at basal, maximal, and spare capacity endpoints (**b**). Addition of control group without EAE in Study 2 illustrates ARE gene induction in placebo-treated mice resulting from EAE that is further increased by CAW treatment (**c**). Addition of control group also demonstrates reduction of OCR values in placebo cohort due to EAE, which were returned to normal with CAW treatment (**d**). *Hmox1,* heme oxygenase 1; *Gclc*, glutamate-cysteine ligase, catalytic subunit; *NqO1*, *NAD(P)H* dehydrogenase-quinone oxidoreductase 1; *Nrf2*, nuclear factor (erythroid-derived 2)-like 2. * *p* < 0.05, ** *p* < 0.01, *** *p* < 0.001.

CAW-treated mice demonstrated a trend towards increased ARE gene expression in the cerebral cortex over placebo-treated mice in Study 1 (Figure 3a, Table 2), and this difference was also attained in Study 2 (Figure 3b). The addition of the control group without EAE in Study 2 highlighted the expected induction of ARE gene expression in placebo animals because of EAE. CAW further increased this compensatory response to inflammatory stress.

CAW-treated mice demonstrated greater cortical mitochondrial respiratory activity than placebo-treated animals in Study 1 across the study profile (*F*(1,2) = 42.64, *p* < 0.001) and between groups at each study’s endpoint (Figure 3b, Table 2). Likewise, mitochondrial respiratory activity was influenced by group in Study 2 (*F*(2,27) = 3.601, *p* = 0.041), with Tukey’s post hoc for CAW vs. placebo reaching significance at the maximal endpoint (*p* = 0.0005, Figure 3d). Notably, the decreased oxygen consumption rates (OCR) in placebo-treated EAE animals compared to healthy controls were fully restored by CAW.

## 3. Discussion

In two independent studies, we found that treatment with CAW, a water extract of the botanical *C. asiatica*, increased both cortical ARE gene expression and cortical mitochondrial respiratory activity and reduced spinal cord inflammation in mice with EAE, an induced inflammatory model of MS. The effects of CAW specifically on microglial activation were equivocal; aside from reduced levels of anxiety on day 12 (increased time in the center of the open field), there were no effects on behavioral performance, on clinical motor disability, or on demyelination.

The novelty of our study is its demonstration that the beneficial increases in ARE gene expression and the restoration of normal mitochondrial respiratory activity using the inflammatory EAE mouse model are consistent with the benefits found in studies using dementia and aging mouse models [13,23]. Specifically, CAW-induced increases in ARE gene expression were observed in the frontal cortex and hippocampi of 5XFAD Aβ-amyloid mice (a transgenic model used in Alzheimer’s research) and in 18-month (old) C57BL/6J mice [23]. Mitochondrial respiratory activity also improved in 5XFAD Aβ-amyloid mice in response to CAW treatment, but there was no change in the mitochondrial respiration of WT mice after CAW treatment, suggesting a ceiling effect for this biomarker [23]. Indeed, CAW-treated EAE mice in our study demonstrated restoration of mitochondrial respiration that met, but did not exceed, the bioenergetic profile of control mice.

Interpretations of the effects of CAW treatment on neuroinflammation and microglial activation based on our study are made with caution. Decreased monocyte infiltration of spinal cords, as seen in the CAW-treated mice with EAE, is generally considered beneficial in MS drug discovery. However, the decrease noted in our study was not accompanied by reductions in EAE clinical disability. Mitochondrial dysfunction begins early in EAE and, interestingly, is not limited to the CNS [24]. Mitochondrial dysfunction and oxidative stress are postulated to both cause and result from inappropriate microglial activation in MS, although tissue damage caused by EAE inflammation limits the evaluation of these pathologies. Newer models may circumvent this issue and could be considered for future evaluations of CAW in EAE [21].

The lack of behavioral changes in response to CAW treatment in our studies has several possible explanations. While mice induced with EAE are reported to display behavioral alterations, a common concern is that the motor disability from EAE obstructs the assessment of behavioral performance [25]. However, given that behavioral performance did not differ between early symptomatic testing and final testing, motor weakness was less likely to have confounded our results. Other possible explanations are that the young mice in our studies may not have manifested behavioral changes from EAE; the behavioral tests chosen were not sensitive to EAE-induced injury; the short treatment duration precluded the detection of behavioral benefits; or the CAW dose, route of administration, or number of mice treated were insufficient to detect treatment responses [13]. Finally, it is possible that CAW simply does not affect behavioral performance in EAE, but may have other clinical correlations to the improved biomarkers.

The mechanisms by which CAW exerts effects on ARE gene expression, mitochondrial respiration, and neuroinflammation were not elucidated in the present study. Normalization of acetylcholinesterase activity, modulation of ERK1/2 and Akt pathways that regulate arborization and synaptogenesis, and inhibition of phospholipase A2 are all postulated mechanisms [14,26,27]. Phytochemical analyses of CAW in other studies suggest roles for specific components such as triterpenes, caffeoylquinic acids, asiatic acid, asiaticoside, and madecassoside that cause neuroprotective and anti-inflammatory effects [28]. While future studies using phytochemical analyses may pinpoint the active constituents, it is also possible that the interactions between constituents are important to the therapeutic effects.

The limitations of this study include the differences in treatment durations prior to sacrifice in Studies 1 and 2, preventing the combination of data for analysis. The use of a single dose of CAW prevented the evaluation of dose-dependent effects, as seen in a non-EAE model [13]. Cognitive tests were not assessed in this study for reasons previously discussed, which precludes conclusions about CAW’s effects on EAE cognition. The effects of older age were also not tested, but could be added to future investigations. Other studies have addressed the confounder of motor weakness when testing cognition in EAE, techniques which could be used in future evaluations [25]. FACS analyses were performed using equipment that precluded the creation of histograms, a correctable issue for future studies. The use of qualitative and not quantitative analysis of fluoromyelin staining is a limitation—fluoromyelin staining is difficult to quantify given the variability in lesion location and the degree of demyelination induced by EAE and, therefore, it is typically analyzed by qualitative methods [29,30]. Although some authors have developed quantitative methods for evaluating demyelination, these strategies use immunolabelling with myelin markers and require analysis of the whole spinal cord instead of sections, do not allow for other IHC analysis, and have their own intrinsic limitations [31]. Another potential limitation of the study is that the ARE-regulated gene expression and mitochondrial respiratory activity measurements were conducted in cortical synaptosomes and not directly in cortical neurons. However, in addition to being more technically feasible, the synaptosome preparation reflects the functional outcomes of neural cell body changes in these measurements, which likely has a bigger impact on neural connections and the behavioral consequences of those connections. Synaptic injury is of increasing interest in MS and has been shown to be affected in the EAE model [32,33].

In summary, the inflammatory EAE model of MS recapitulates the CAW-induced increases in ARE gene expression and mitochondrial respiratory activity seen in aging and dementia models, and also demonstrates decreased inflammation. Benefits for the pathophysiology common across neurodegenerative disorders suggest similar therapeutic effects. Further exploration is warranted to determine clinical correlations to the striking improvements in these biomarkers of oxidative health in an effort find treatments for neuropsychiatric symptoms of MS.

## 4. Materials and Methods

### 4.1. CAW Production, Analysis, and Administration

The CAW used in this study was produced at the Oregon Health & Science University (OHSU) BENFRA Botanical Dietary Supplements Research Center, as previously described [34]. Briefly, *Centella asiatica* dried herb (batch number X20090016) was obtained through the company Oregon’s Wild Harvest (Redmond, OR, USA) from a supplier in India (Organic India Private Ltd., Lucknow, India). Voucher samples are stored at OHSU (BEN-CA-6) and in the herbarium at Oregon State University (OSC-V-265416). The plant material was authenticated as *C. asiatica* at the BENFRA Center by comparing its thin layer chromatographic profile with American Herbal Pharmacopoeia botanical reference material for *C. asiatica* (# 5626) and by DNA fingerprinting. This authenticated *C. asiatica* material was used to prepare CAW, as previously described [34]. Briefly, *C. asiatica* herb (4 kg) was extracted by boiling in water (50 L) for 90 min. The extract was filtered to remove solid plant debris, the liquid filtrate was divided between several aluminum trays and frozen. The frozen extract was lyophilized in three separate runs to yield a dry residue (CAW; total weight from three batches, 820 g). Voucher samples of the three dried CAW batches (BEN-CAW-7, 8, and 9) are stored at the BENFRA center.

### 4.2. CAW Phytochemical Analysis

Studies by our group and others indicate that triterpene and caffeoylquinic acid components are involved in CAW’s neurotropic, cognition-enhancing, antioxidant activities, as well as its ability to improve mitochondrial function [35]. The analysis of triterpenes and caffeoylquinic acids in BEN-CAW-8, using liquid chromatography coupled to multiple reaction monitoring mass spectrometry (LC-MRM-MS), was performed by the BENFRA Center and has been described in detail [34]. Briefly, chromatographic separation was performed using an Intersil Phenyl-3 column, eluting with a seven-minute gradient of methanol in water, both containing 0.1% formic acid. This system allowed for the resolution of three mono-caffeoylquinic acids, five di-caffeoylquinic acids, and four triterpenes, which were detected using negative electrospray ionization and MRM-MS. The relevant MRM transitions used and the % *w*/*w* content in BEN-CAW-8 for each of these compounds were as follows: 3-caffeoylquinic acid (353/191; 0.72%), 4-caffeoylquinic acid (353/173; 0.30%), 5-caffeoylquinic acid (353/191; 0.34%), 1,3-dicaffeoylquinic acid (515/179; 0.25%), 1,5-dicaffeoylquinic acid (515/191; 0.39%), 3,4-dicaffeoylquinic acid (515/173; 0.23%), 3,5-dicaffeoylquinic acid (515/191; 0.20%), 4,5-dicaffeoylquinic acid (515/173; 0.22%), asiatic acid (533; 487; 0.08%), madecassic acid (549/503; 0.14%), asiaticoside (1003/957; 1.46%), madecassoside (1019/973; 3.45%).

### 4.3. CAW and Placebo Administration to Mice

CAW was dosed at 500 mg/kg/day using two drops of CAW solution (125 mg/mL) dissolved in 5% sucrose/phosphate buffered saline (PBS) solution. The placebo consisted of an equal volume of 5% sucrose/PBS solution. The placebo and CAW solutions were stored at −20 °C until use. A fresh vial of placebo or CAW treatment was thawed for use each day to avoid repeated freeze-thawing, then loaded into individual pipette tips for oral administration. The dose chosen was equal to that used successfully in a dose-escalation study in 5XFAD dementia animals [13].

### 4.4. Animals

Because of changes to the dosing schedule and the addition of a control group without EAE, the experiments are reported separately.

Study 1: Eight to twelve-week-old female C57BL/6J mice (*n* = 20; Jackson Labs, Sacramento, CA, USA) were divided into two cohorts and housed in mixed treatment groups of four per cage. Mice were kept in the Animal Resource Facility at the Portland VA Medical Center on a 12 h light/dark cycle with access to food and water ad libitum. To induce moderate EAE, each cohort was inoculated by subcutaneous injection on a single day with 0.2 mL of an emulsion containing 200 μg complete Freund’s adjuvant containing heat-killed Mycobacterium tuberculosis strain H37Ra and 200 μg mouse myelin oligodendrocyte glycoprotein (MOG) peptide35-55 homogenate. Mice received intraperitoneal booster injections of 75 ng and 200 ng Pertussis toxin on days zero and two, respectively [36]. Starting at an EAE clinical disability score of two, mice received daily treatment with either CAW or placebo in a one-to-one distribution. Saline perfusion followed by euthanasia by cervical dislocation occurred after 14 days of treatment on two pre-planned days. Half of the mice underwent immediate brain dissection for harvesting of the cerebral cortex, while the other half underwent brain and spinal cord dissection for subsequent FACS.

Study 2: Four-month-old female C57BL/6J mice (*n* = 30; Jackson Labs, Sacramento, CA, USA) were divided into three equally sized mixed treatment groups and matched by initial weight. Mice were housed at the OHSU animal facility. Control mice received sham inoculations and boosters with saline followed by placebo treatment (5% sucrose in PBS). The remaining two groups received EAE induction as described above, followed by either placebo treatment or CAW (dosed as in Study 1), assigned one-to-one based on weight. Treatments began on day six after inoculation each morning and continued for 14 days, including the day of euthanasia (day 20). Several animals required supplemental saline treatments due to weight loss. Following euthanasia, which was performed by cervical dislocation, all mice immediately underwent brain and spinal cord dissection, with half of all brains harvested to collect the cerebral cortex, while lumbar spinal cords were prepared for IHC.

### 4.5. Weight, EAE Clinical Disability, and Behavioral Testing

Weight was recorded at baseline and daily in Study 1 and at baseline and starting on day five (pretreatment) in Study 2. EAE clinical disability based on motor weakness was scored daily by the same technician using a scale from zero (no disability) to six (moribund requiring euthanasia). Intermediate scores are limp tail (one), moderate hindlimb weakness or mild ataxia (two), splayed hindlimbs affecting gait (three), one hindlimb substantially or completely paralyzed (four), both hindlimbs paralyzed (five). A final CDI score was calculated as the sum of the daily scores over the treatment period (numerical integration of the EAE score curve over the entire experiment to represent total disease load).

Behavioral performance, including exploratory activity and anxiety levels, was assessed in the open field test. The open field consisted of a well-lit square (L 40.6 × W 40.6 × H 40.6 cm) with a central light intensity of 100 lux. Mice were allowed to explore the open field for 10 min during two consecutive days. Two adjacent arenas were used simultaneously for testing. The enclosures were cleaned with 0.5% acetic acid between trials. The performance of mice was tracked using Ethovision 15 XT software. The performance aspects analyzed were total Distance Moved (general activity), Center Duration (anxiety), Latency to the First Center Entry, and Center Frequency (Study 2 only). Open field testing occurred on the final study day for Study 1 and on days 5 (pretreatment), 12 (early disability), and day 20, the final day, in Study 2.

### 4.6. FACS and IHC

Inflammation, including microglial activation, was evaluated in the dissected brains and spinal cords in Study 1 via FACS analyses. Leukocytes were labelled for mononuclear cells (CD74+) and microglia (CD45loCD11b+ and CD45hiCD11b+) using monoclonal antibodies, as previously described [37]. Due to expected low cell counts, tissues from animals within the same treatment groups were pooled prior to FACS testing.

For IHC and tissue staining, tissues were fixed in 4% paraformaldehyde, freeze-embedded, serially sectioned at a thickness of 10 μm on a cryostat (Leica, Wetzler, Germany), and placed on glass slides. Tissues were then immunostained with either anti-CD3 antibodies (BD Pharmingen, San Francisco, CA, USA, 1:300; to label inflammation) or anti-Iba1 antibodies (Wako Life Sciences, Mountain View, CA, USA, 1:300; to label activated microglia) along with Hoechst 3342 (to label cell nuclei), followed by detection with fluorescence-labeled secondary antibodies, as previously described [38]. Myelin was detected using fluoromyelin (Thermo Fisher Scientific, Hillsboro, OR, USA) with the goal of making a qualitative assessment of the influence of CAW on demyelination [39].

### 4.7. ARE Gene Expression and Mitochondrial Respiratory Activity

Gene expression was quantified in cortical synaptosomes. Synaptosomes were freshly isolated from one half of the cortical tissue from the left hemisphere using Syn-per reagent (Thermo Scientific #87793, Waltham, MA, USA) as per the manufacturer’s protocol. Total RNA was collected via Tri-Reagent extraction (Molecular Research Center, Cincinnati, OH, USA) and cDNA was produced via reverse transcription using the Superscript III First Strand Synthesis Kit (Invitrogen, Carlsbad, CA, USA) per manufacturers’ instructions. Relative gene expression was measured using Taqman primers and probes (*Nfe2l2*; *Nrf2*-Mm00477784_m1), NAD(P)H dehydrogenase-quinone oxidoreductase 1 (*Nqo1*; Mm001253561_m1), glutamate-cysteine ligase, catalytic subunit (*Gclc;* Mm00802655_m1), heme oxygenase 1 (HMOX1, *Ho-1*; Mm00516005_m1), and reagents (TaqMan Gene Expression Master Mix) from Applied Biosystems (Foster City, CA, USA) with normalization to glyceraldehyde-3-phosphate dehydrogenase (*Gapdh*; hs02758991_g1 (Applied Biosystems, Foster City, CA, USA)) expression. qPCR was performed using a StepOne Plus Machine (Applied Biosystems, Foster City, CA, USA) and analyzed using the delta-delta Ct method. All groups were normalized to the control mice.

Mitochondrial respiratory activity was assessed in cortical synaptosomes using the Seahorse XFe96 Analyzer (Agilent, Santa Clara, CA, USA). The total protein concentration of the synaptosomal preparation for each mouse was determined using a bicinchoninic acid (BCA) assay. A total of 10 ug of synaptosomal protein diluted in 25 uL mitochondrial assay solution (MAS; 70 mM sucrose, 220 mM mannitol, 10 mM KH_2_PO_4_, 5 mM MgCl_2_, 2 mM HEPES, 1 mM EGTA, 0.2% BSA) was plated in each well of a polyethylenimine (PEI)-coated 96-well Seahorse plate (5–6 replicate wells for each animal) and the plate was centrifuged at 1200 g for 1 h at 4 °C. A total of 155 μL of Agilent Seahorse XF DMEM Medium pH 7.4 (Part # 103575-100) supplemented with 1 mM pyruvate, 2 mM glutamine, and 10 mM glucose was plated in each well of the 96-well plate.

Mitochondrial OCR was then assessed using the MitoStress kit (Agilent #103015-100) as previously described [40]. Briefly, OCR was measured under varying conditions. After three initial baseline measurements of OCR, the adenosine triphosphate (ATP) synthase inhibitor oligomycin (2 μM) was added and three subsequent measurements were taken. Next, an electron transport chain accelerator, p-trifluoromethoxy carbonyl cyanide phenyl hydrazone (FCCP) at 2 μM, was added and three measurements of maximal respiration were taken. Finally, the mitochondrial inhibitors rotenone (0.5 μM) and antimycin (0.5 μM) were added and three final measurements were taken. Spare capacity was calculated by subtracting the average of the basal measurements from the average of the three maximal measurements following FCCP administration.

### 4.8. Statistical Analyses

Study 1: Differences in the mean changes in weight, CDI scores, behavioral tests, and ARE gene expression were compared between groups by *t*-test. For the Latency to First Center entry, animals that never entered the center were given latency equal to the full trial length (600 s). Mitochondrial function was compared using repeated measures analysis of variance (ANOVA) across the study profile with Tukey’s post hoc test and by *t*-tests between groups at the basal, maximal, and spare capacity endpoints. Because the FACS analyses used pooled tissues (3 mice per treatment group contributed to each sample tested) due to expected low CNS cell numbers, the statistical error could not be calculated; instead, results were qualitatively compared.

Study 2: Mean changes in weight were compared between groups by repeated measures ANOVA, with Tukey’s post hoc analysis being employed for group differences in daily weight starting at day 5 and cognitive behavioral scores at days 12 and 20. CDI scores were compared by *t*-test between CAW- and placebo-treated mice. ARE gene expression groups were compared using ANOVA with Dunnett’s post hoc tests for pairwise comparison. For mitochondrial respiration, repeated measures ANOVA was calculated across the profile and a separate ANOVA was used for each endpoint (i.e., one for basal respiration, another for maximal respiration, etc.) with Tukey’s post hoc for pairwise comparison. IHC results for the CD3 and Iba1 counts were analyzed with *t*-tests between placebo- and CAW-treated EAE mice. Comparisons of the demyelination of the spinal cord sections between groups were qualitative for reasons described in Section 3. All group differences, compared with *t*-tests, were first examined graphically for normality using normal probability plots. The significance for all tests was set at *p* < 0.05.

## Figures and Tables

**Table 1:** Fluorescence-activated cell sorting analysis of pooled spinal cord samples of monocyte expression (CD74+) resting (CD11b + CD45low) and activated (CD11b + CD45hi) microglia in brains and spinal cords of mice with experimental autoimmune encephalomyelitis (EAE) treated with *Centella asiatica* or placebo. Results presented as percentages (%) of total cell count. CT45int: intermediate expression.

	Brain (*n* = 3)		Spinal Cord (*n* = 3)	
	Placebo	*Centella asiatica*	Placebo	*Centella asiatica*
CD74+ %	4.0	4.2	19.7	15.0
CD11b + CD45low %	54.1	58.3	17.6	19.0
CD11b + CD45int %	17.0	24.8	26.1	26.7
CD11b + CD45hi %	24.9	16.7	46.6	51.5
Total Cell Count	~2.4 × 10^6^	~2 × 10^6^	~4.3 × 10^6^	~4.8 × 10^6^
Viability %	62.0	66.0	60.0	62.0

CD, cluster of differentiation; int, intermediate; hi, high.

**Table 2:** Antioxidant response element (ARE) gene expression and mitochondrial respiration (oxygen consumption rate, OCR) in cortical tissues from Studies 1 and 2. Comparisons in Study 1 between CAW-treated and placebo-treated mice with experimental autoimmune encephalomyelitis used *t*-tests with Tukey’s post hoc. For Study 2, comparisons used Dunnett’s pairwise comparisons for ARE gene expression and Tukey’s post hoc for OCR. ANOVA results are presented in text. Sample sizes were *n* = 9 per group for Study 1 and *n* = 10 per group for Study 2, with exceptions individually noted.

Outcome	Study 1		Study 2		
(Mean, SEM)	Placebo	CAW	Control	Placebo	CAW
ARE: *Nrf2* fold induction	1 ± 0.0.24	1.66, 0.31 *p* = 0.15	1 ± 0.23 vs. CAW, *p* = 0.002	2.12 ± 0.43 vs. control, *p* = 0.34	3.99 ± 0.90 vs. placebo, *p* = 0.07
ARE: *Hmox1* fold induction	1 ± 0.30	1.42, 0.39 *p* = 0.43	1 ± 0.14 vs. CAW, *p* = 0.002	1.63 ± 0.24 vs. control, *p* = 0.42	2.90 ± 0.58 vs. placebo, *p* = 0.047
ARE: *Nqo1* fold induction	1 ± 0.29	1.31, 0.26 *p* = 0.46	1 ± 0.29 vs. CAW, *p* = 0.0003	2.03 ± 0.35 vs. control, *p* = 0.27	4.09 ± 0.71 vs. placebo, *p* = 0.013
ARE: *Gclc* fold induction	1 ± 0.24	1.95, 0.48 *p* = 0.13	1 ± 0.17 vs. CAW, *p* = 0.0001	1.88 ± 0.26 vs. control, *p* = 0.12	3.20 ± 0.46 vs. placebo, *p* = 0.016
OCR-basal (pmol O_2_/min)	133.1 ± 4.4 *n* = 3	156.8, 6.4 *n* = 4, *p* = 0.04	105.20 ± 2.99 vs. CAW, *p* = 0.99	90.08 ± 3.55 vs. control, *p* = 0.03	105.56 ± 4.98 vs. placebo, *p* = 0.18
OCR-maximal (pmol O_2_/min)	156.4 ± 9.2 *n* = 3	190.28, 5.7 *n* = 4, *p* = 0.03	138.90 ± 5.16 vs. CAW, *p* = 0.96	112.60 ± 3.10 vs. control, *p* = 0.001	140.82 ± 6.33 vs. placebo, *p* = 0.0005
OCR-spare capacity (pmol O_2_/min)	47.8 ± 5.0 *n* = 3	64.8, 2.8 *n* = 4, *p* = 0.03	33.62 ± 6.97 vs. CAW, *p* = 0.98	22.52 ± 4.87 vs. control, *p* = 0.38	35.26 ± 5.29 vs. placebo, *p* = 0.28

ARE, antioxidant response element gene; CAW, water extract of *C. asiatica*; *Gclc*, glutamate-cysteine ligase, catalytic subunit; *Hmox1*, heme oxygenase 1; *Nqo1*, NAD(P)H dehydrogenase-quinone oxidoreductase 1; *Nrf2*, nuclear factor (erythroid-derived 2)-like; OCR, oxygen consumption rate; SEM, standard error of mean.

## Data Availability

Data are available upon request to the corresponding author.
